# Preclinical Evaluation of CRISPR-Edited CAR-NK-92 Cells for Off-the-Shelf Treatment of AML and B-ALL

**DOI:** 10.3390/ijms232112828

**Published:** 2022-10-24

**Authors:** Guillermo Ureña-Bailén, Jérôme-Maurice Dobrowolski, Yujuan Hou, Alicia Dirlam, Alicia Roig-Merino, Sabine Schleicher, Daniel Atar, Christian Seitz, Judith Feucht, Justin S. Antony, Tahereh Mohammadian Gol, Rupert Handgretinger, Markus Mezger

**Affiliations:** 1 Department of Hematology and Oncology, Children’s Hospital, University Hospital Tuebingen, 72076 Tuebingen, Germany; 2 MaxCyte Inc., Rockville, MD 20850, USA; 3 Cluster of Excellence iFIT (EXC2180) “Image-Guided and Functionally Instructed Tumor Therapies”, University of Tübingen, 72074 Tuebingen, Germany

**Keywords:** NK-92, CD19-CAR, CD276-CAR, leukemia, AML, B-ALL, CRISPR-Cas9 knock-out, CBLB, NKG2A, TIGIT

## Abstract

Acute myeloid leukemia (AML) and B-cell acute lymphocytic leukemia (B-ALL) are severe blood malignancies affecting both adults and children. Chimeric antigen receptor (CAR)-based immunotherapies have proven highly efficacious in the treatment of leukemia. However, the challenge of the immune escape of cancer cells remains. The development of more affordable and ready-to-use therapies is essential in view of the costly and time-consuming preparation of primary cell-based treatments. In order to promote the antitumor function against AML and B-ALL, we transduced NK-92 cells with CD276-CAR or CD19-CAR constructs. We also attempted to enhance cytotoxicity by a gene knockout of three different inhibitory checkpoints in NK cell function (CBLB, NKG2A, TIGIT) with CRISPR-Cas9 technology. The antileukemic activity of the generated cell lines was tested with calcein and luciferase-based cytotoxicity assays in various leukemia cell lines. Both CAR-NK-92 exhibited targeted cytotoxicity and a significant boost in antileukemic function in comparison to parental NK-92. CRISPR-Cas9 knock-outs did not improve B-ALL cytotoxicity. However, triple knock-out CD276-CAR-NK-92 cells, as well as CBLB or TIGIT knock-out NK-92 cells, showed significantly enhanced cytotoxicity against U-937 or U-937 CD19/tag AML cell lines. These results indicate that the CD19-CAR and CD276-CAR-NK-92 cell lines’ cytotoxic performance is suitable for leukemia killing, making them promising off-the-shelf therapeutic candidates. The knock-out of CBLB and TIGIT in NK-92 and CD276-CAR-NK-92 should be further investigated for the treatment of AML.

## 1. Introduction

Acute leukemia comprises a group of heterogenous, progressive clonal disorders driven by genetic mutations in blood progenitor cells. Such genetic changes ultimately induce an unrestrained potential for self-renewal together with a developmental arrest of affected progenitor cells at a specific stage of their differentiation. The resulting immature cells (blasts) are likely to invade the bone marrow and the reticulo-endothelial system along with other extramedullary areas, thereby inhibiting various functions of the organism and eventually leading to death if not properly treated [1]. Among the various subsets of acute leukemia, acute lymphocytic leukemia (ALL) is the most frequent malignant disorder in children, whereas acute myeloid leukemia (AML) is the most prevalent blood disorder in adults [2,3].

In recent years, chimeric antigen receptor (CAR)-based immunotherapies have emerged for the treatment of leukemia. Effective CAR therapies against B-ALL have been approved by the FDA in late 2017 [4]. Nevertheless, there are key limitations that can compromise the efficacy of the treatment. Drawbacks, such as immunosuppression, graft-versus-host disease (GvHD) and host-versus-graft effect (HvG), can be overcome by further genetic enhancement of CAR effector cells [5]. Iatrogenic effects such as cytokine release syndrome (CRS) and neurotoxicity are major hurdles in CAR-T cell therapies, which can be prevented by selecting a different suitable CAR carrier such as natural killer (NK) cells [6]. Additional concerns arise with regard to the highly costly, long-time manufacturing process of CAR cells derived from primary immune cells, where convenient off-the-shelf therapies can aid [6,7]. Taking these aspects into consideration, we have developed CAR-NK-92 cells and tested their cytotoxicity against different leukemic cell lines to assess their therapeutic efficacy against AML and B-ALL. Further CRISPR-Cas9-induced genetic improvements were also considered. The increasing number of clinical and preclinical studies reporting improved anticancer cytotoxicity after gene knock-out or antibody blockade of inhibitory checkpoints sets the grounds for a wider range of treatment options in the future [5,8,9,10,11,12,13,14,15,16,17]. However, critical questions may arise as to which strategy is more effective for treating a certain type of cancer. We have considered a side-by-side comparison of the knock-out of three important inhibitory checkpoints in NK cell effector function (CBLB, NKG2A, TIGIT) to assess their antileukemic efficacy in the presence or absence of the CAR. In addition, triple knock-outs were studied to corroborate potential additive or synergistic effects in the modulation of cytotoxic performance.

## 2. Results

### 2.1. Ligand Profile Characterization of Leukemia Cell Lines

**Figure 1:**
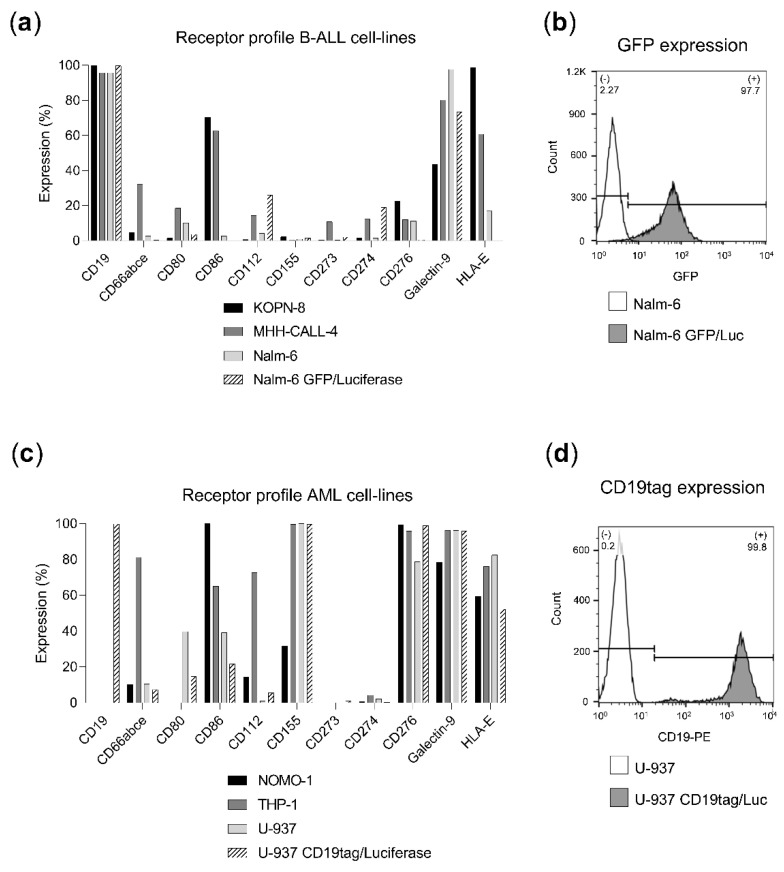
Ligand profile characterization of leukemia cell lines. Percentage of ligand or GFP-expressing cells was determined by flow cytometry. (**a**) Ligand expression in B-ALL cell lines (KOPN-8, MHH-CALL-4, Nalm-6 and Nalm-6 GFP/Luc). (**b**) GFP expression of Nalm-6 GFP/Luc (grey) in comparison to non-transduced Nalm-6 (white). (**c**) Ligand expression in AML cell lines (NOMO-1, THP-1, U-937 and U-937 CD19tag/Luc). (**d**) CD19 expression of U-937 CD19tag/Luc (grey) in comparison to non-transduced U-937 (white).

AML and B-ALL cell lines were analyzed by flow cytometry to determine the expression of each target antigen (CD19 and CD276) as well as relevant ligands for inhibitory receptors including CTLA-4 (CD80 and CD86), NKG2A (HLA-E), PD-1 (CD273 & CD274), TIGIT (CD112 and CD155) and TIM-3 (CD66a and Gal-9) (Figure 1) [18]. B-ALL cell lines (KOPN-8, MHH-CALL-4, Nalm-6, Nalm-6 GFP/Luc) showed a high CD19 expression (expressed in 95–100% of the cells, Figure 1a) and a modest expression of inhibitory ligands, being Gal-9 the most abundantly expressed (50–100%, Figure 1a). Transgenic Nalm-6 GFP/Luc cell line reported a high expression of GFP (97.7%, Figure 1b). On the other hand, AML cell lines (NOMO-1, THP-1, U-937, U-937 CD19tag/Luc) expressed high levels of CD276 (80–100%, Figure 1c). AML cell lines exhibited a wider expression of inhibitory molecules, including CD155, Gal-9 and HLA-E (30–100%, 80–100% and 60–80%, respectively, Figure 1c). Transgenic U-937 CD19tag/Luc cell line showed high levels of CD19 (99.8%, Figure 1d).

### 2.2. Receptor Profile Characterization of Effector Cell Lines

**Figure 2:**
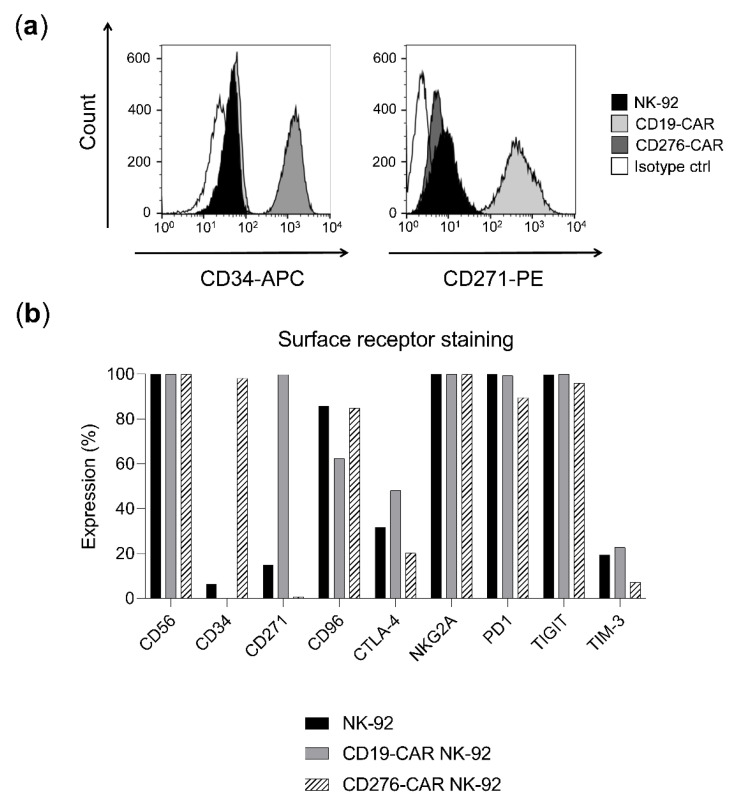
Receptor profile characterization of NK-92 cell lines. Percentage of receptor-expressing cells was determined by flow cytometry. (**a**) Expression of CAR reporter genes (CD34 and CD271) in NK-92 (black), CD19-CAR NK-92 (light grey), CD276-CAR NK-92 (dark grey) and NK-92 isotype control (white). (**b**) Receptor expression in NK-92 (black), CD19-CAR (grey) and CD276-CAR-NK-92 cell lines (white).

To verify the presence of the CAR in the transduced NK-92 cell lines, the staining of a reporter gene included in each CAR construct was studied: CD34 expression was correlated with CD276-CAR levels and expression of CD271 was an indicator of CD19-CAR expression (98% CD34 expression in CD276-CAR-NK-92 and 100% CD271 expression in CD19-CAR-NK-92, respectively, Figure 2a,b). The inhibitory checkpoints of CAR and parental NK-92 cells were further analyzed, showing a similar expression profile across all cell lines (Figure 2b). High levels of PD1, NKG2A and TIGIT were observed (90–100%, 99–100% and 95–100%, respectively, Figure 2b).

### 2.3. CAR-NK-92 Outperforms Parental NK-92 Antileukemic Activity

**Figure 3:**
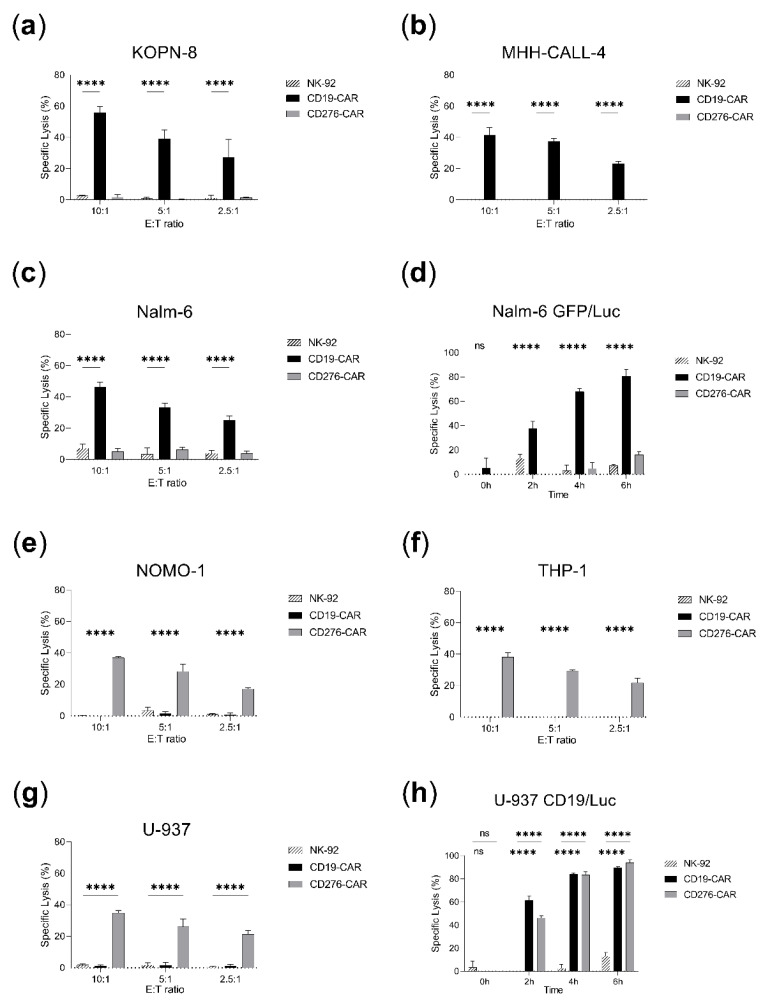
Cytotoxicity assays comparing NK-92 and CAR-NK-92 antileukemic performance. Specific lysis is shown as mean ± SD (*n* = 3). Calcein assays were incubated for 2 h and several effector-to-target (E:T) ratios were employed: 10:1, 5:1, 2.5:1. Luciferase assays were performed over a time span of 6 h in 5:1 E:T ratio. (**a**) KOPN-8 calcein assay. (**b**) MHH-CALL-4 calcein assay. (**c**) Nalm-6 calcein assay. (**d**) Nalm-6 GFP/Luc luciferase assay. (**e**) NOMO-1 calcein assay. (**f**) THP-1 calcein assay. (**g**) U-937 calcein assay. (**h**) U-937 CD19tag/Luc luciferase assay. **** *p* < 0.0001, ns, non-significant (*p* > 0.05).

Both parental and CAR-NK-92 cells were tested in vitro with leukemia cell lines. Different effector-to-target ratios were investigated in calcein release assays and a time-course cytotoxicity study was analyzed in luciferase assays (Figure 3). CD19-CAR-NK-92 showed significantly improved specific lysis in calcein assays (40–60% against KOPN-8, MHH-CALL-4 and Nalm-6) in relation to their parental counterpart (up to 10% specific lysis) (Figure 3a–c). A similar effect was observed in luciferase assays, where CD19-CAR-NK-92 cytotoxicity (85% against Nalm-6 GFP/Luc after 6 h) greatly outperformed non-transduced NK-92 (8% specific lysis after 6 h) (Figure 3d). This enhanced cytolytic activity was also induced by CD276-CAR-NK-92 in AML cell lines (less than 5% of specific lysis in calcein assays for parental NK-92 vs. 30–40% in CD276-CAR-NK-92; 16% specific lysis in luciferase assays for NK-92 after 6 h vs. 95% for CD276-CAR-NK-92, Figure 3e–h). The cytotoxic performance of both CAR cell lines was specific and targeted as no antileukemic activity was observed in absence of their ligand (Figure 3a–h). CD276-CAR-NK-92 cytotoxicity against B-ALL was identical or decreased compared to parental NK-92 (Figure 3a–d). A similar activity could be observed for CD19-CAR against AML cell lines except for the transgenic luciferase-expressing U-937 cell line due to the high expression of CD19tag (up to 90% specific lysis after 6 h, Figure 3h).

### 2.4. CRISPR-Cas9 Knock-Out of CBLB, NKG2A and TIGIT

**Figure 4:**
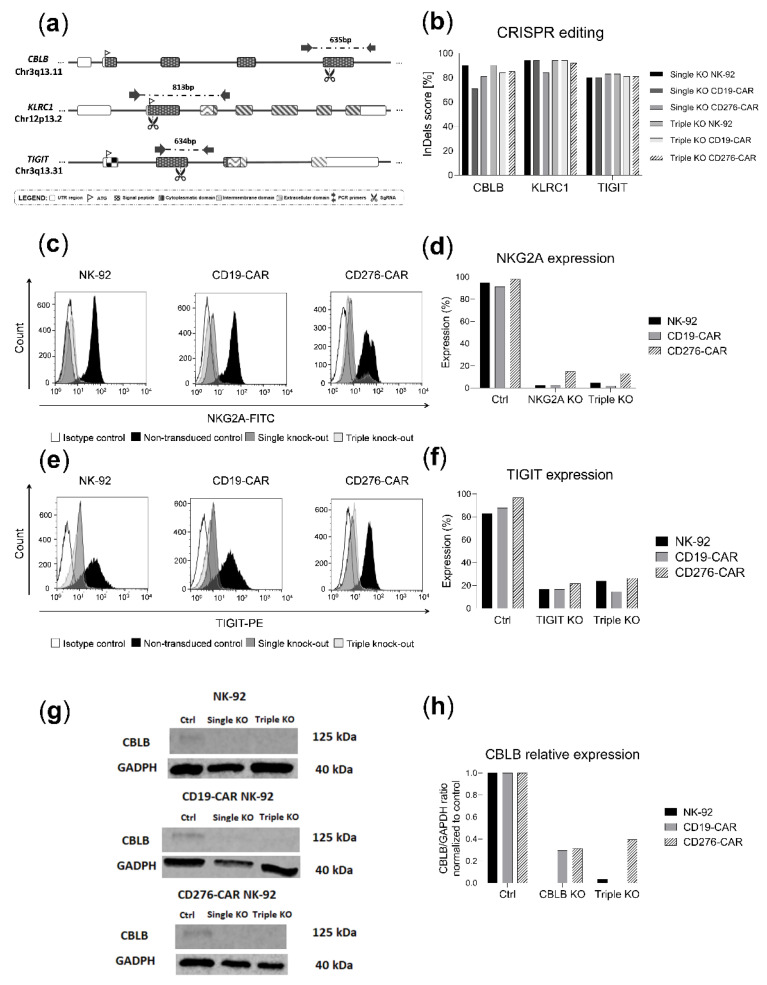
CRISPR-Cas9 editing of NK-92 and CAR-NK-92 cell lines. (**a**) Schematic illustration showing the cutting sites of CBLB, NKG2A and TIGIT gRNAs in their corresponding genes as well as depicting primers and PCR amplicon sizes for InDel analyses. (**b**) InDel score for CBLB, NKG2A and TIGIT in single and triple knock-out cell lines. (**c**) NKG2A histogram expression in parental cell line (black), single knock-out cell line (dark grey), triple knock-out cell line (light grey) and isotype control (white). (**d**) NKG2A protein levels in single and triple knock-out cell lines. (**e**) TIGIT histogram expression in parental cells (black), single knock-out cells (dark grey), triple knock-out cells (light grey) and isotype control (white). (**f**) TIGIT protein levels in single and triple knock-out cells. (**g**) Immunoblot of CBLB and GADPH in single and triple knock-out cells. (**h**) Normalized CBLB/GADPH ratio of band densitometry readings in single and triple knock-out cells.

To further enhance the antileukemic activity of NK-92 and CAR-NK-92, three inhibitory molecules (CBLB, NKG2A, TIGIT) were knocked out by CRISPR-Cas9. Additionally, a sequential triple knock-out of all targets was generated in the same cell-line. The employed single-guide RNAs (sgRNAs) aimed at exonic regions of the aforementioned targets (Figure 4a). Sequencing results revealed high InDel (Insertion or Deletion) efficiency for all three targets (CBLB: 71–90%, NKG2A: 84–94%, TIGIT: 80–83%, Figure 4b) and flow cytometry analyses confirmed the knock-outs at the protein level for NKG2A and TIGIT (85–100% reduction for NKG2A and 60–75% for TIGIT, Figure 4c–f). Immunoblots showed a marked reduction in CBLB protein levels following CRISPR-Cas9 treatment, consistent with the high knock-out efficacy achieved (60–100% reduction in protein expression, Figure 4g,h).

### 2.5. Evaluation of the Effect of Inhibitory Checkpoint Knock-Out in Parental and CD19-CAR-NK-92 B-ALL Killing Assays

**Figure 5:**
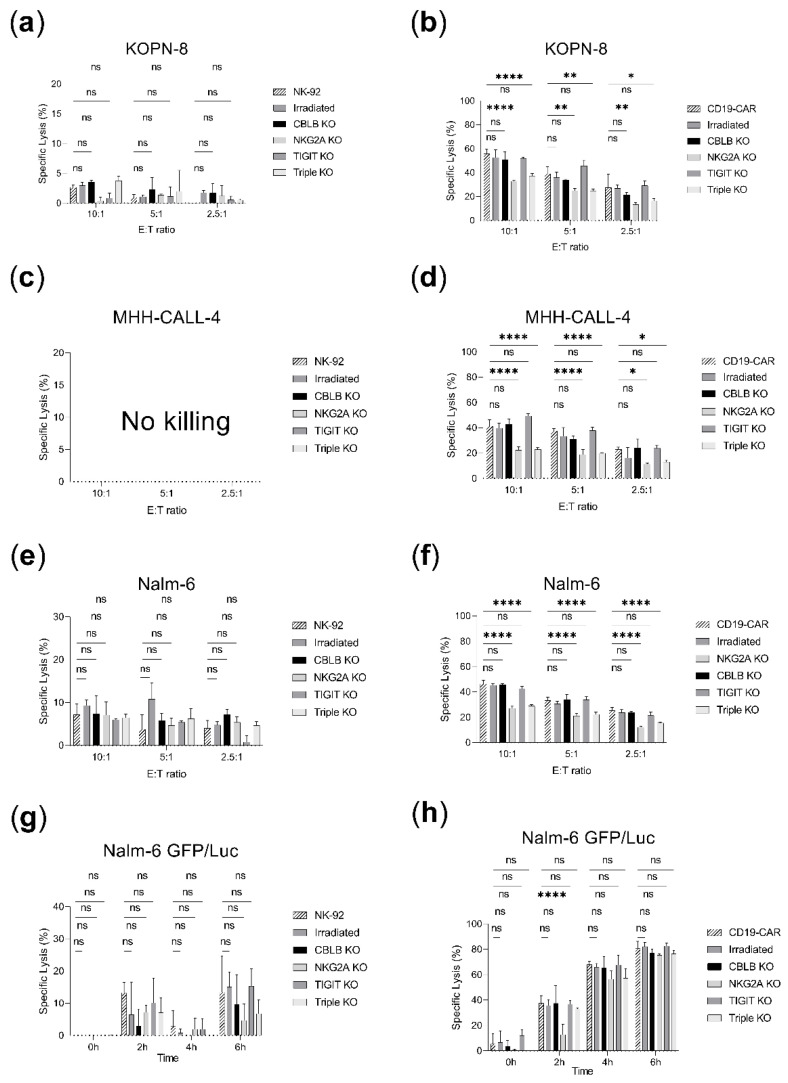
Cytotoxicity assays in B-ALL cell-lines comparing the cytotoxicity of parental NK-92 and CD19-CAR-NK-92 vs. irradiated, CBLB, NKG2A, TIGIT or triple knock-out cells. Specific lysis is shown as mean ± SD (*n* = 3). Calcein assays were incubated for 2 h and several effector-to-target (E:T) ratios were employed: 10:1, 5:1, 2.5:1. Luciferase assays were studied for a time span of 6 h at 5:1 E:T ratio. (**a**) NK-92 cell lines vs. KOPN-8 calcein assay. (**b**) CD19-CAR-NK-92 cell lines vs. KOPN-8 calcein assay. (**c**) NK-92 cell lines vs. MHH-CALL-4 calcein assay did not display cytotoxicity in the tested conditions (no killing). (**d**) CD19-CAR-NK-92 cell lines vs. MHH-CALL-4 calcein assay. (**e**) NK-92 cell lines vs. Nalm-6 calcein assay. (**f**) CD19-CAR-NK-92 cell lines vs. Nalm-6 calcein assay. (**g**) NK-92 cell lines vs. Nalm-6 GFP/Luc luciferase assay. (**h**) CD19-CAR-NK-92 cell lines vs. Nalm-6 GFP/Luc luciferase assay. * *p* < 0.05; ** *p* < 0.01; **** *p* < 0.0001; ns, non-significant (*p* > 0.05).

The generated effector cell lines were tested along with their parental counterpart in cytotoxicity assays (Figure 5). CBLB and TIGIT knock-out CD19-CAR-NK-92 showed comparable killing rates to unedited CD19-CAR-NK-92 (40–60% in 10:1 E:T ratios in calcein assays and up to 85% in luciferase assays, Figure 5b,d,f,h). Unexpectedly, the killing efficacy of CD19-CAR-NK-92 was reduced with NKG2A or triple knock-out against KOPN-8, MHH-CALL-4 and Nalm-6 but analysis at later time points (4–6 h) in luciferase assay against Nalm-6 GFP/Luc revealed similar performance to CD19-CAR-NK-92 (80.8 ± 4.5% for CD19-CAR, 75 ± 0.7% for NKG2A knock-out and 76 ± 2.3 for triple knock-out, Figure 5h). Overall, CBLB, NKG2A and TIGIT knock-out CD19-CAR cell lines did not improve the antileukemic activity of CD19-CAR cells (Figure 5b,d,f,h). Similarly, no enhancement of the effector function could be observed in the knock-out NK-92 cell lines (Figure 5a,c,e,g). Irradiation of parental or CD19-CAR-NK-92 did not decrease the killing efficacy (Figure 5a–h).

### 2.6. Evaluation of the Effect of Inhibitory Checkpoint Knock-Out in Parental and CD276-CAR-NK-92 AML Killing Assays

**Figure 6:**
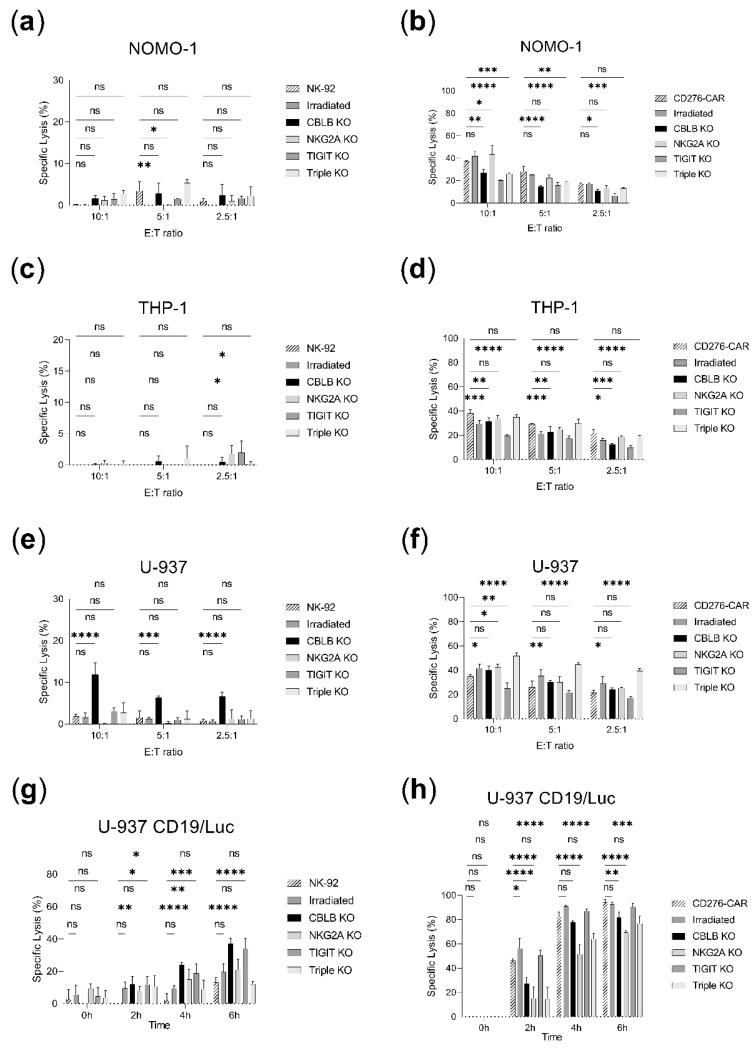
Cytotoxicity assays in AML cell lines comparing parental NK-92 and CD276-CAR-NK-92 vs. irradiated, CBLB, NKG2A, TIGIT or triple knock-out cells. Specific lysis is shown as mean ± SD (*n* = 3). Calcein assays were incubated for 2 h and several effector-to-target (E:T) ratios were employed: 10:1, 5:1, 2.5:1. Luciferase assays were studied for a time span of 6 h at 5:1 E:T ratio. (**a**) NK-92 cell lines vs. NOMO-1 calcein assay. (**b**) CD276-CAR-NK-92 cell lines vs. NOMO-1 calcein assay. (**c**) NK-92 cell lines vs. THP-1 calcein assay. (**d**) CD276-CAR-NK-92 cell lines vs. THP-1 calcein assay. (**e**) NK-92 cell lines vs. U-937 calcein assay. (**f**) CD276-CAR-NK-92 cell lines vs. U-937 calcein assay. (**g**) NK-92 cell lines vs. U-937 CD19tag/Luc luciferase assay. (**h**) CD276-CAR-NK-92 cell lines vs. U-937 CD19tag/Luc luciferase assay. * *p* < 0.05; ** *p* < 0.01; *** *p* > 0.001; **** *p* < 0.0001; ns, non-significant (*p* > 0.05).

The antileukemic effect of CRISPR-modified NK-92 and CD276-CAR-NK-92 cells was assessed in cytotoxicity assays in AML cell lines (Figure 6). A significant boost of the cytotoxicity was observed for CBLB knock-out NK-92 against U-937, as well as CBLB and TIGIT knock-out NK-92 against U-937 CD19tag/Luc after 6 h (20–30% or 2.5 to 3-fold improvement, Figure 6e,g). CBLB knock-out in NK-92 also exhibited significant improvement against NOMO-1 and Nalm-6 in 5:1 and 2.5:1 E:T ratios, respectively, but no benefit is observable in higher E:T ratios (Figure 6a,c). In contrast, CD276-CAR-NK-92 triple knock-out seemed to increase antileukemic activity in U-937 (20% increase in 10:1 E:T ratios, Figure 6f), but not U-937 CD19tag/Luc where the specific lysis was slightly lower (15% decrease at 6 h, Figure 6h). CBLB and TIGIT knock-outs underperformed against NOMO-1 and THP-1 (15% or 1.5 to 2-fold decrease, Figure 6b,d) but exhibited comparable killing to CD276-CAR vs. U-937 and its luciferase-expressing clone (25% in 5:1 E:T ratio and 90% at 6 h, respectively, Figure 6f,h). Killing rates were neither improved with NKG2A knock-out and remained comparable to parental CD276-CAR-NK-92 (40%, 20% and 25% specific lysis, respectively, Figure 6b,d,f) or lower (20–30% decrease at 6 h, Figure 6h). Irradiation of parental or CD276-CAR-NK-92 demonstrated similar cytotoxicity to its non-irradiated counterpart (Figure 6a–h).

## 3. Discussion

CD19-CAR-NK-92 cell-based treatment was previously reported to exert potent and specific cytotoxicity in B-cell precursor cell lines and leukemic blasts [19]. There have been no previous studies on CD276-CAR-NK-92 cells for the treatment of AML, but their efficacy has been tested against other malignancies such as neuroblastoma or melanoma. Furthermore, earlier studies in T cells proved the effectiveness of CD276-CAR against AML cell lines and mouse models [20,21,22,23]. These observations are consistent with the results observed in the present study, where both CD19-CAR and CD276-CAR NK-92 cells exhibit a targeted and strong cytotoxic effect against B-ALL and AML cell lines, respectively, in comparison to the parental cell lines (*p* < 0.0001, Figure 3). This enhancement of the antileukemic performance, together with the unlimited source material and ease of expansion, makes CAR-NK-92 cells promising and affordable candidates for the off-the-shelf treatment of leukemia.

However, one of the main drawbacks of NK-92-based immunotherapy lies on their limited persistence in the host and thereby gradual loss of anticancer function [6]. This limitation originates from the necessary irradiation of the effector cells to ensure a safe therapeutic application. In the current study, both CD19 and CD276-CAR functionalities were not affected after 10Gy irradiation (up to 6 h of incubation, Figure 5 and Figure 6). Nonetheless, a substantial loss of cytotoxicity in the days following the infusion is well described [24]. To overcome the loss of therapeutic efficacy due to the reduced lifespan and subsequent cytotoxicity decline of irradiated NK-92 cells, sequential transfusions of readily available effector cells are considered in the treatment scheme. Notwithstanding, this dosage strategy increases the risk of anti-HLA antibody formation against NK-92 and can negatively affect the outcome of the therapy [25,26]. Hence, it would be crucial to exert the maximum cytotoxic effect as quickly as possible to take advantage of the full antileukemic potential of each NK-92 infusion and reduce the number of doses.

Inhibitory ligands expressed by the leukemic blasts or their microenvironment can inhibit or delay the antileukemic response [27,28]. Since the expression of several inhibitory ligands was observed in both AML and B-ALL cell lines (Figure 1a,c), disrupting inhibitory checkpoints expressed on NK-92 could potentially improve their effector function. CBLB, NKG2A and TIGIT, three inhibitory checkpoints the suppression of which has been shown to boost antileukemic treatment, were selected as knock-out targets. CBLB ablation previously demonstrated cytotoxicity enhancement in placental stem-cell-derived NK cells against a HL-60 leukemia mouse model and was known to be expressed in the NK-92 cell line [9,29]. Furthermore, NKG2A blockade has been shown to induce tumor cell death in a leukemia mouse model [30]. Its expression was reported in the effector cell lines (Figure 2b) and its main ligand, HLA-E, was highly expressed in most of the target cell lines used in this work (Figure 1a,c). Finally, TIGIT was considered since its blockade in combination with other blocking antibodies resulted in enhanced NK-92 cytotoxicity against AML and its ligands CD112 and CD155 expressed in several target cell lines (Figure 1a,c) [31]. TIM-3 was initially considered as well, due to its relevance in AML immunosuppression and prominent expression of Gal-9 in the target cells but was eventually not evaluated due to its low expression in the effector NK-92 cell lines (Figure 2b) [32].

We also considered multiplexing knock-outs to favor the balance towards NK activation, which is highly dependent on the milieu of activatory versus inhibitory signals [18,27,33]. Previous research suggests that more effective strategies can be achieved when targeting several inhibitory checkpoints at the same time [31]. Accordingly, the triple knock-out of the target genes was pursued in a step-by-step approach consisting of a single target knock-out at a time, separated by at least two weeks of culture expansion to ensure proper cell recovery prior to a new transfection. This sequential method was preferred to one-shot multiplexing to avoid potential translocations or chromosomic rearrangements generated by multiple DSBs’ induced at the same time [34].

Very high rates of protein knock-outs were attained for all targets (Figure 4). These efficient transfection protocols can be easily transferred to other targets of interest in NK-92 immunotherapy. Despite successful CRISPR-induced modifications, our results show no consistent improvement of the single or multiple knock-out of these inhibitory checkpoints in combination with CD19 or CD276-CAR constructs (Figure 5 and Figure 6). This observation is in accordance with our previous study of NKG2A knock-out in CD276-CAR-NK-92 against melanoma, where no enhancement of the functionality could be reported [20]. Only in very specific set-ups, such as triple knock-out CD276-CAR versus U-937, did the knock-out approach seem to be significantly advantageous (Figure 6f). More importantly, we have reported the underperformance of NKG2A, CBLB, TIGIT or triple knock-out CAR-NK-92 cell lines, in both calcein and luciferase cytotoxicity assays (Figure 5 and Figure 6). Since this effect is not observable in non-transduced NK-92, these unexpected findings could be attributed to loss of cellular fitness after multiple genetic manipulations of CAR-transduced NK-92. In addition, potential CRISPR-derived off-target modifications cannot be excluded. It seems that this negative effect delays the cytotoxic response but does not hamper effective cytotoxicity at later time points, as supported by the observation of a similar performance to CAR-NK-92 at 4–6 h in luciferase assays for most knock-out cell lines (Figure 5h and Figure 6h).

It would seem that the potential benefit of the studied knock-outs in CAR-NK-92 effector function would be small and not worthy of implementation in CAR-NK-92-based antileukemic treatments. However, one of the main limitations of the current study lies in in vitro experimentation. Chamberlain et al. previously reported no benefit of PD-1 knock-out in the anticancer function of tumor-infiltrating T cells (TILs) in cytotoxicity assays [35], despite the fact that its effectiveness has been extensively proven in in vivo pre-clinical research and investigated in clinical trials [8,36]. Similarly, we cannot exclude a favorable outcome of the tested knock-outs under the complexity of in vivo models, where pro-leukemic elements such as the bone microenvironment can play a major role in cancer progression and immunosuppression [37,38]. We have shown significant benefits for CBLB and TIGIT knock-out in NK-92 cells against U-937 AML cell lines (Figure 6e,g). The CBLB knock-out in NK-92 also exhibited significant improvement against NOMO-1 and Nalm-6 in 5:1 and 2.5:1 E:T ratios, respectively, but being the reported cytotoxicity close to the detection level with no benefit presented in higher E:T ratios, we would assume this observation is likely to be an artifact and more time of incubation would be needed to assess the benefit (Figure 6a,c). The improvements observed for CBLB and TIGIT knock-outs in AML could be overshadowed in CD276-CAR-NK-92 cytotoxicity assays by the powerful CAR-driven activation, which almost reached complete lysis of leukemic cells after 6 h of co-culture (Figure 6h). We hypothesize that the CD276-CAR-NK-92 performance might be hindered by more adverse immunosuppressive conditions, where the checkpoint knock-outs could help to restore cytotoxicity or enhance it, as suggested by the significant improvement observed for the triple knock-out CD276-CAR-NK-92 against U-937 cells (Figure 6f). Further studies are needed to assess completely the effect of CBLB and TIGIT knock-outs in CD276-CAR-NK-92 immunotherapy, which are likely to exhibit therapeutic effects in the treatment of AML. We cannot conclude the same for CD19-CAR-NK-92, where the knock-out of CBLB, NKG2A and TIGIT did not improve the cytotoxic performance. Different inhibitory checkpoints should be targeted to enhance B-ALL NK-92-based immunotherapy.

## 4. Materials and Methods

### 4.1. Cell Lines and Culture Conditions

NK-92 parental cells were purchased from the American Type Culture Collection (ATCC) and were cultured with MEM Alpha Medium (1X) + GlutaMAX (Thermo Fisher Scientific, Waltham, MA, USA) supplemented with 20% *v/v* FBS, 1% *v/v* L-glu, 1% *v/v* P/S and 100 U/mL IL-2 (Miltenyi Biotec, Bergisch Gladbach, Germany). AML cell lines (NOMO-1, THP-1, U-937) and B-ALL cell lines (KOPN-8, MHH-CALL-4, Nalm-6) were obtained from the German Collection of Microorganisms and Cell Cultures GmbH (DSMZ, Braunschweig, Germany). Transgenic Nalm-6 GFP/Luciferase and U-937 CD19tag/Luciferase were generated as previously described [39,40]. Leukemia cell lines were cultured with RPMI medium supplemented with 10% *v/v* FBS, 1% *v/v* L-glu, 1% *v/v* P/S (Thermo Fisher Scientific, Waltham, MA, USA) except MHH-CALL-4 which was supplemented with 20% *v/v* FBS.

### 4.2. FACS Staining

Cells were washed with PBS and stained at room temperature using a 1:10 dilution of the indicated antibodies in 100 µL PBS for 10 min. Cells were washed once more to remove antibody excess and flow cytometry data was subsequently acquired with FACSCalibur (BD Biosciences, Franklin Lakes, NJ, USA). Live cells were gated based on forward and side scatter plots. Isotype staining’s were performed as control conditions. Anti-CTLA-4 PE extracellular antibody, anti-Gal-9-PE and their corresponding isotype controls were purchased from Biolegend, San Diego, CA, USA. The rest of the antibodies (anti-CD276-PE, anti-CD80-PE, anti-CD86-APC, anti-CD273-PE, anti-CD274-APC, anti-HLA-E-APC, anti-CD112-PE, anti-CD66abce-APC, anti-CD155-PE, anti-CD19-APC or FITC, anti-PD1-PE, anti-TIM3-APC, anti-NKG2A-FITC, CD56-APC, CD96-APC, TIGIT-PE, CD34-APC, CD271-PE and corresponding isotype controls) were obtained from Miltenyi Biotec, Bergisch Gladbach, Germany. CD19-CAR and CD276-CAR expression on NK-92 cells was determined by CD271 and CD34 marker gene expression, respectively, whereas luciferase expression on target cells was correlated with GFP for Nalm-6 GFP/Luc and CD19 marker expression for U-937 CD19tag/Luc.

### 4.3. CAR Transduction

For CD19-CAR transduction, the 19–28z SFGγ retroviral vector employed in this work (kindly provided by Michel Sadelain (MSKCC) to J.F.) has been previously described [41,42]. Virus and transductions were performed as formerly reported (293Vec-RD114 packaging cells were kindly provided by BioVec Pharma Inc, Québec, Canada) [39]. Second-generation CD276-CAR lentiviral vector and transduction in NK-92 cells were described in an earlier publication [23].

### 4.4. CRISPR-Cas9 Transfection

Three different NK-cell inhibitory checkpoints (CBLB, NKG2A, TIGIT) were disrupted in effector NK-92 and CAR-NK-92 cell lines by the CRISPR-Cas9 system. The employed sgRNAs were previously tested or newly designed using CHOPCHOP v3 software (www.chopchop.cbu.uib.no (accessed on 27 May 2022), Table 1) [43].

*KLRC1* (NKG2A) knock-out was generated by using the Neon transfection system (ThermoFisher, Scientific, Waltham, MA, USA) and knock-out cells were sorted and enriched, as formerly described. [20] CBLB and TIGIT knock-outs were generated employing MaxCyte GTx^TM^ electroporator (MaxCyte Inc, Rockville, MD, USA). V3 Cas9 and sgRNA (IDT, Coralville, IA, USA) were incubated at a molar ratio of 1:2 (1.5 µmol to 3 µmol) for 15 min to favor RNP complexation. Two and a half million cells were washed and resuspended in 50 µL of MaxCyte^®^ electroporation buffer, then mixed with the RNP and electroporated in R-50x3 processing assemblies using the NK-4 electroporation protocol (MaxCyte, Rockville, MD, USA). Cells were seeded in pre-warmed 12-wellplates after electroporation and were incubated for 30 min in the incubator. Next, fresh medium without antibiotics and interleukins was added at 2 × 10^6^ cells/mL. After four hours, a culture medium with 2X IL-2 (200 U/mL) was added to have a final concentration of 1 × 10^6^ cells/mL. For the generation of triple knock-out, NKG2A knock-out cells were expanded for two weeks before transfecting CBLB RNP with the same MaxCyte protocol as described in this section. The double knock-out cells were expanded again for two weeks and finally transfected with TIGIT RNP using the MaxCyte protocol (MaxCyte, Rockville, MD, USA).

### 4.5. Assessment of CRISPR-Cas9-Induced Knock-Out

On day 5 post-electroporation, the CRISPR-modified cells were harvested for DNA isolation with the NucleoSpin Tissue kit following the manufacturer’s instructions (Macherey-Nagel, Düren, Germany) and were employed in PCR reactions to amplify the target region (primer sequences and PCR protocols described in Table S1). Samples were cleaned up of remaining reagents with QIAquick PCR purification kit (Qiagen, Hilden, Germany) and were sequenced by Sanger-sequencing (Eurofins Genomics, Ebersberg, Germany). CRISPR-induced insertions and deletions were analyzed by ICE v3 software (www.ice.synthego.com (accessed on 17 May to 2 August 2022), Synthego, Redwood, CA, USA). [44] NKG2A and TIGIT knock-outs were assessed by flow cytometry as aforementioned whereas CBLB knock-out was analysed by Western blot.

### 4.6. CBLB Western Blot

Three to five million cells were resuspended in RIPA Lysis and Extraction Buffer (ThermoFisher, Scientific, Waltham, MA, USA) supplemented with 1X Halt™ Protease and Phosphatase Inhibitor Cocktail (ThermoFisher, Scientific, Waltham, MA, USA) and incubated on ice for 20 min. The soluble fraction was recovered after 10 min of centrifugation at 10,000× *g* 4 °C. Protein concentration was determined by standard Bradford assay. A total of 20 µg of protein was loaded in a Mini-PROTEAN TGX gel (Bio-Rad, Hercules, CA, USA) and separated by electrophoresis. The gel was transferred to a Midi format 0.2 µm PVDF membrane using Trans-Blot Turbo Transfer System (Bio-Rad, Hercules, CA, USA). The membrane was blocked with EveryBlot Blocking Buffer (Bio-Rad, Hercules, CA, USA) and incubated for 1 h at room temperature (RT) or overnight at 4 °C with rabbit anti-CBLB (Cell Signaling Technologies, Danvers, MA, USA, clone: D3C12) diluted at 1:500 and rat anti-GAPDH (Biolegend, San Diego, CA, USA, clone: W17079A) diluted at 1:1000 in blocking buffer. After washing, the membrane was incubated for 1 h at room temperature with IRDye 800CW goat anti-rat and IRDye 680RD goat anti-rabbit (LI-COR Biosciences, Lincoln, NE, USA) at 1:15,000 in a blocking buffer. The membrane was developed using LI-COR Odyssey Fc and band intensity was quantified with Image Studio 4.0 (LI-COR Biosciences, Lincoln, NE, USA).

### 4.7. Calcein Release Assay

Calcein solution (Calcein AM, Biolegend, San Diego, CA, USA) was prepared at 1 µg/µL in DMSO. Target cells were washed and resuspended in PBS at 10^6^ cells/mL, then incubated at 37 °C 5% CO_2_ in the incubator with 10 µL of calcein solution per mL of cell suspension for 1 h with gentle swirls every 10 min. Next, target cells were washed three times with assay medium (RPMI 10% FBS) to remove calcein excess. The target cell solution was prepared at 10^5^ cells/mL in assay medium and 100 µL was added to a 96 well-plate. 100 µL of effector cells were added to the target cells at indicated ratios in technical triplicates. Two controls were prepared in the same plate: spontaneous lysis (SL, target cells in assay medium) and maximum killing (MK, target cells in assay medium with Tryton X-100). The plate was placed in the incubator for 2 h before taking fluorometric measurements of the supernatant in a TECAN Spark reader (TECAN, Männedorf, Switzerland) to measure calcein release. Effector-specific lysis (ESL) was calculated as below:

ESL = (measured value − SL)/(MK − SL) × 100(1)

### 4.8. Luciferase Assay

Nalm6-expressing Luc-GFP and U937 CD19tag/Luc served as target cells for luciferase cytotoxicity assay protocols. In brief, 1 × 10^4^ target cells were resuspended in 100 µL of assay medium (RPMI 10% FBS) and co-cultured with 5 × 10^4^ effector cells in 50 µL of assay medium for different time points in the incubator (0–6 h) and in technical triplicates. D-luciferin (Gold-Bio, St. Louis, MO, USA) was dissolved in pure water to a final concentration of 15 mg/mL and stored at −20 °C. To generate the working assay solution, a stock aliquot was mixed 1:4 in RPMI 10% FBS and 50 µL were added to each assay well (final volume 200 µL) immediately before the luminescence measurement in TECAN Spark reader (TECAN, Männedorf, Switzerland). Effector-specific lysis (ESL) was calculated according to spontaneous lysis (SL, target cells in assay medium with luciferin) and maximum killing controls (MK, target cells in assay medium with Tryton X-100 and luciferin) using Formula (1).

### 4.9. Data Analysis

All graphical and statistical analyses were performed with GraphPad Prism 9 software (GraphPad Software Inc., San Diego, CA, USA). Two-way ANOVA analyses were performed for gaussian distributed datasets. Multiple non-parametric *t*-tests were performed when the Shapiro–Wilk test for normal distribution was not passed. Flow cytometry data were analyzed using FlowJo software (FlowJo LLC., BD Biosciences, Franklin Lakes, NJ, USA).

## Figures and Tables

**Table 1:** List of sgRNAs employed in this study.

Target Gene	gRNA Nucleotide Sequence	Reference
*CBLB*	TAATCTGGTGGACCTCATGA	Guo et al. [9]
*KLRC1* (NKG2A)	GGTCTGAGTAGATTACTCCT	Grote et al. [20]
*TIGIT*	ACCCTGATGGGACGTACACT	Own design (CHOPCHOP)

## Data Availability

The data presented in this study are available from the corresponding author upon reasonable request.

## Supplementary Materials

The following supporting information can be downloaded at: https://www.mdpi.com/article/10.3390/ijms232112828/s1.
