# Supplementary material for: Preclinical Evaluation of CRISPR-Edited CAR-NK-92 Cells for Off-the-Shelf Treatment of AML and B-ALL
Source: Int J Mol Sci. 2022 Oct 24;23(21):12828. doi: 10.3390/ijms232112828 (PMC9655234; doi:10.3390/ijms232112828)
Supplement: Supplementary file 1 [file ijms-23-12828-s001.zip › ijms-1960416-supplementary.pdf]

Table S1: Primers and PCR protocols employed for sequencing and InDel analyses in this work

Target	Forward primer	Reverse primer	PCR protocol	Amplicon size
CBLB	TGATAGCCTAGGACTGTTTGAGAGAA	GTTATCAGATGCTGTGAGCCTGG	initial denaturation of 95 C for 2 min followed by 40 cycles of 40 s at 95 C, 40 s at 55 C and 1 min at 68 C	635bp
NKG2A	TACTCGTTCTCCACCTCACC	TAACGTGAAAATTCCCCTTGTAATC		813bp
TIGIT	GGCACAATAGAAACAACGGGG	TATGACCTCATCAACTGGTCTTCC		634bp
